# Investigation on the Thermal Decomposition Behavior of Molybdenum Trioxide Precursor

**DOI:** 10.3390/ma18010165

**Published:** 2025-01-03

**Authors:** Xiao Zhang, Pengfa Feng, Xuyang Liu, Chunyang Bu, Kuaishe Wang, Hang Qu

**Affiliations:** 1 School of Metallurgy and Engineering, Xi’an University of Architecture and Technology, Xi’an 710055, China; 2 Jinduicheng Molybdenum Co., Ltd., Xi’an 710077, China; 3 Shaanxi “Four Bodies and One Union” University-Enterprise Jiont Research Center for Advanced Molybdenum-Based Functional Materials, Xi’an 710077, China; 4 National and Local Joint Engineering Research Center for Functional Materials Processing, Xi’an University of Architecture and Technology, Xi’an 710055, China

**Keywords:** MoO_3_ precursor, MoO_3_, thermal decomposition temperature, thermal decomposition kinetics

## Abstract

The ultrafine MoO_3_ powders were prepared by the combination of centrifugal spray drying and calcination in this work. The thermal decomposition behavior of the spherical precursor was studied. The phase constituents, morphologies, particle size, and specific surface areas of MoO_3_ powders were characterized at different temperatures. It is found that the decomposition of the precursor is subjected to five stages, and forms different intermediate products, including (NH_4_)_8_Mo_10_O_34_, (NH_4_)_2_Mo_3_O_10_, (NH_4_)_2_Mo_4_O_13_, h-MoO_3_, and the final product α-MoO_3_. Moreover, the decomposition rate equation is established based on the thermal decomposition kinetic parameters of the precursor. With an increase in decomposition temperature, the morphology changes from unclear boundary particles to dispersed flake particles, and the flaky particles exhibit larger sizes, higher crystallinity, and better dispersion, which can be attributed to the mass transfer of gaseous MoO_3_ products. Additionally, the MoO_3_ particle size decreases progressively, and the specific surface area increases and then decreases. At 500 °C, it can achieve ultrafine flaky MoO_3_ powder with the size of thick sheets, with a thickness of about 300 nm and a length of about 1–3 μm. This research can offer an innovative strategy for preparing ultrafine MoO_3_ powder.

## 1. Introduction

Generally, the transition metal oxide molybdenum trioxide (MoO_3_) typically presents in the forms of the thermodynamically stable orthorhombic phase α-MoO_3_, the non-thermodynamically stable monoclinic phase β-MoO_3_, and the hexagonal phase h-MoO_3_ and MoO_3-II_ [1,2], due to the varying arrangements of the basic unit [MoO_6_] octahedra; thus, it exhibits excellent properties in photoluminescence, electrochromism, photocatalytic degradation, and gas sensitivity, which are widely used in lithium-ion batteries, gas sensors, and catalysis [3,4,5,6,7]. Additionally, it can serve as a primary raw material for the production of molybdenum powder and molybdenum products. Currently, several studies revealed that the morphology of MoO_3_ remarkably affects the morphology and particle size of molybdenum powder [8,9]. Moreover, ultrafine MoO_3_ possesses high theoretical specific capacitance, specific surface to volume ratio, stable thermal and mechanical properties, which is widely used as electrode material in hybrid capacitors and supercapacitors [10,11,12].

Traditionally, the MoO_3_ powder is fabricated by the thermal decomposition of ammonium molybdate. However, the prepared MoO_3_ powder displays coarse particles and poor size uniformity, which cannot satisfy the requirements for preparing ultrafine molybdenum powder. Thus far, a few methods are utilized for preparing ultrafine molybdenum powder [13,14,15,16,17,18,19]. Din et al. [16] prepared the MoO_3_ micro-belts with an average thickness of 600 nm and a length of 5 μm using (NH_4_)_6_Mo_7_O_24_.4H_2_O and nitric acid as raw materials by hydrothermal reaction. Wang et al. [18] synthesized MoO_3_ nanorods with a dimeter of 10–33 nm on different substrates by the thermal chemical vapor deposition method at low pressure. Choi et al. [19] utilized an ultrasonic spray pyrolysis technology to fabricate ultrafine MoO_3_ particles, and also found that Mo_4_O_11_ is formed simultaneously at the spray pyrolysis temperature of above 700 °C. Though ultrafine MoO_3_ powder can be achieved by these methods, it generally requires higher temperature, higher pressure, or longer time, which is not conducive to mass production. Consequently, it is necessary to seek a novel technology to fabricate ultrafine molybdenum oxide powder. Centrifugal spray drying, a rapid drying method, has huge advantages in manufacturing ultrafine powder, such as simplicity, convenience, a high yielding efficiency, and a high recovery rate. In the centrifugal spray drying process, the pre-prepared slurry (solution or suspension) is pumped into the atomizer by a peristaltic pump, and then atomized at a high speed to form a lot of droplets. Once the small droplets encounter the hot gas flow in the chamber, liquid droplets evaporate into solid particles quickly [20,21,22]. The powders fabricated display spherical morphology, along with a narrow particle size distribution and uniform composition [22,23,24,25,26]. Moreover, there are also no residues and wastewater during the process.

In this work, ultrafine MoO_3_ powder was prepared by the combination of centrifugal spray drying and calcination. The thermal decomposition behavior and intermediate products were studied at different temperatures, the morphologies, particle size, and specific surface area of MoO_3_ powder were characterized, and the formation mechanism of ultrafine MoO_3_ was discussed.

## 2. Experimental Section and Characterization

**Figure 1:**
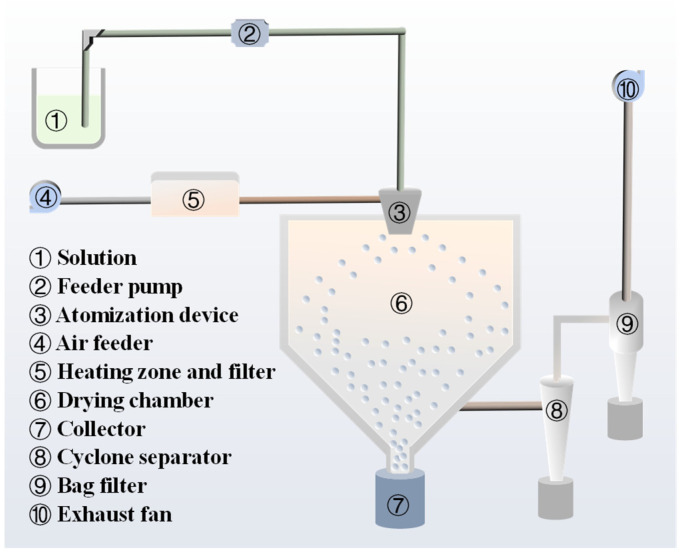
Schematic diagram of centrifugal spray drying.

Ammonium dimolybdate (ADM, 99.9%, Jinduicheng Molybdenum Co., Ltd., Xi’an, China) and ammonia solution (AR, Sinopharm Chemical Reagent Co., Ltd., Shanghai, China) were added into the deionized water to synthesize a transparent solution by continuous mechanical stirring. Afterwards, the solution was used to fabricate the molybdenum trioxide precursor by centrifugal spray drying. The precursor synthesis method is original, and the details of the preparation process are as follows. Firstly, the filtered air was introduced by air feeder, followed by heating until the inlet temperature and outlet temperature, respectively, reached 240 °C and 110 °C. Secondly, the pre-synthesized solution was pumped to the centrifugal atomizer at a frequency of 27 Hz. Subsequently, the solution was sprayed into tiny droplets and dried in the hot air flow. Finally, the MoO_3_ precursor powders were obtained. The schematic diagram of the pilot-scale facility of the centrifugal spray drying is illustrated in Figure 1.

The thermal decomposition of the MoO_3_ precursor was investigated with a thermal analyzer (STA449C + QMS 403 (MS), Netzsch, Selb, Germany). The heating rate of 5, 10, 15, 20 °C/min, sample sizes of 4–5 mg, and flowing air (50 mL /min) were used during the measurements.

A tubular furnace with water cooling zones at both ends was used to carry out the thermal decomposition experiments of the MoO_3_ precursor. Overall, 100 g of the MoO_3_ precursor was placed into a corundum boat (150 mm × 40 mm × 20 mm), followed by heating at a desired temperature (1 or 2 temperatures were selected in each mass loss interval based on the TG curve, 400 °C, 450 °C, 500 °C, 550 °C) for 1.5 h in the air atmosphere. Finally, the reaction production was pushed to the cooling zone of the furnace and cooled to room temperature.

The morphologies of precursor and reaction production powders were characterized by a field emission scanning electron microscope (FE-SEM, Tescan Clara, Brno, Czech Republic). The phase constituents of the powders were identified by X-ray diffraction (XRD-7000, Shimadzu, Kyoto, Japan), and the precursor was dried at 65 °C for 2 h in the drying box prior to the measurements. The particle size distributions of powders were determined by a laser particle size analyzer (Mastersizer 2000, Malvern Instruments, Malvern, UK) with ethyl alcohol (AR).

The specific surface areas of the MoO_3_ powders were measured by N_2_ adsorption at 77 K on a surface area and pore size analyzer (NOVE touch LX4, Quantachrome Instruments, Boynton Beach, FL, USA). The powders were degassed at 200 °C for 4 h under vacuum for removing residual moisture and volatiles before the measurements. The specific surface areas were calculated by the evaluation of the linearized form of the BET Equation based on adsorption data obtained in the *P*/*P*_0_ (N_2_) range from 0.05 to 0.30 [27]:

(1) $$\frac{1}{V((P_{0}/P)-1)}=\frac{1}{V_{m}C}+\frac{C-1}{V_{m}C}(\frac{P}{P_{0}})$$

where *P* is the pressure of N_2_ and *P*_0_ is its saturation vapor pressure, *V* is the amount of N_2_ adsorbed per unit mass, *V_m_* is the amount of saturated adsorption in a monolayer, *P*/*P*_0_ is the relative pressure of adsorbed N_2_, and *C* is a constant.

## 3. Results and Discussion

### 3.1. Characterization of MoO_3_ Precursor

**Figure 2:**
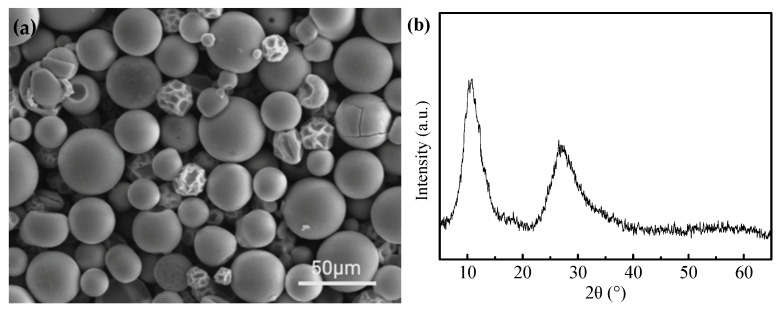
Morphology (**a**) and XRD pattern (**b**) of MoO_3_ precursor.

Figure 2a displays the morphologies of the MoO_3_ precursor. As seen from Figure 2a, most MoO_3_ particles present hollow and spherical morphology along with a smooth surface. The formation of hollow particles can be ascribed to the rapid drying of the droplets when encountering hot air during the centrifugal atomizing process. This also results in the presence of residue water inside the shell. Under the action of capillarity, the residual water subsequently migrates to the surface of the outer shell, thus bringing about the formation of hollow particles.

Figure 2b shows the X-ray diffraction pattern of the MoO_3_ precursor. It is apparent that the prepared precursor displays an amorphous ammonium molybdate, which can be attributed to the rapid drying of centrifugally atomized droplets and no sufficient time to be crystallized.

### 3.2. Thermal Decomposition Behavior of MoO_3_ Precursor

**Figure 3:**
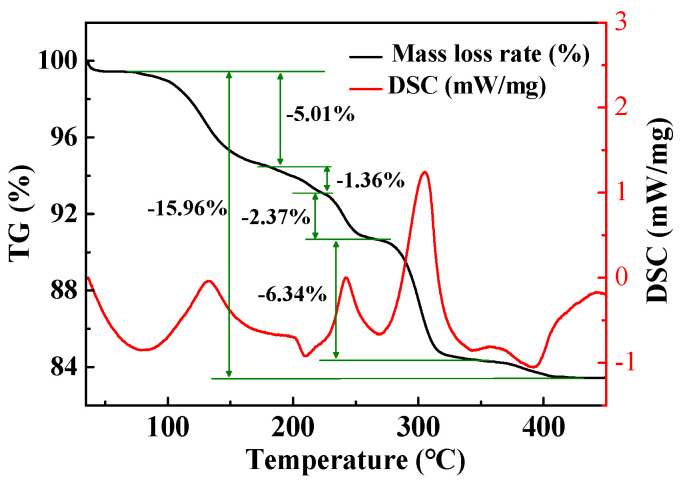
TG/DSC curves for the decomposition of MoO_3_ precursor.

To clarify the thermal decomposition behavior of the MoO_3_ precursor, the TG/DSC-MS analysis was conducted on the resultant precursor, and the TG/DSC curves are presented in Figure 3. Distinctly, five mass losses occur for the MoO_3_ precursor during the thermal decomposition process, and the total mass loss rate is 15.96%. From the DSC curve, three obvious endothermic peaks occur at 132.7 °C, 242.5 °C, and 305.5 °C, while two slight exothermic peaks appear at 210.3 °C and 391.7 °C.

**Figure 4:**
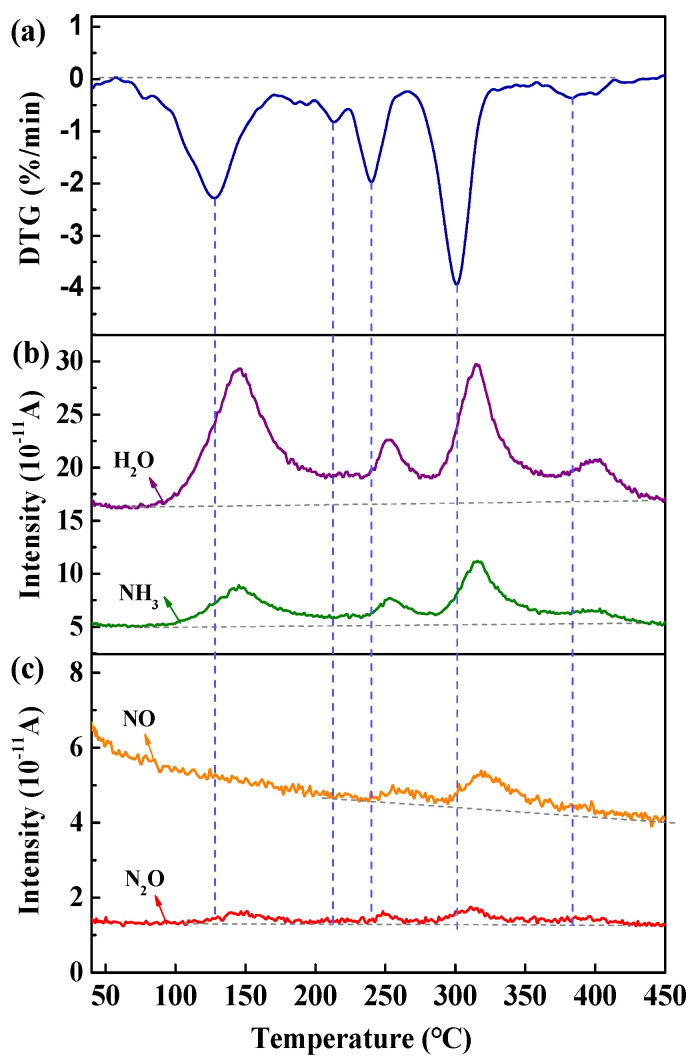
DTG (**a**), mass spectrometric of H_2_O, NH_3_ (**b)** and of NO, N_2_O (**c**) curves for the thermal decomposition of MoO_3_ precursor.

Figure 4 displays the DTG and mass spectrometric curves of the MoO_3_ precursor during the thermal decomposition process. There are five peaks in the DTG curve in Figure 4a. From Figure 4b, it can also be seen that the mainly released gasses matched to *m*/*z* = 17 and 18 are NH_3_ and H_2_O, respectively. Though NH_3_ and H_2_O peaks are similar to that in the DTG curve, their intensities are slightly different, implying that NH_3_ and H_2_O are produced simultaneously during the decomposition process of the MoO_3_ precursor. In addition, besides NH_3_ and H_2_O, NO and N_2_O (*m*/*z* = 30 and 44) are also detected as seen in Figure 4c, which are caused by the incomplete burning of the released NH_3_ [28].

**Figure 5:**
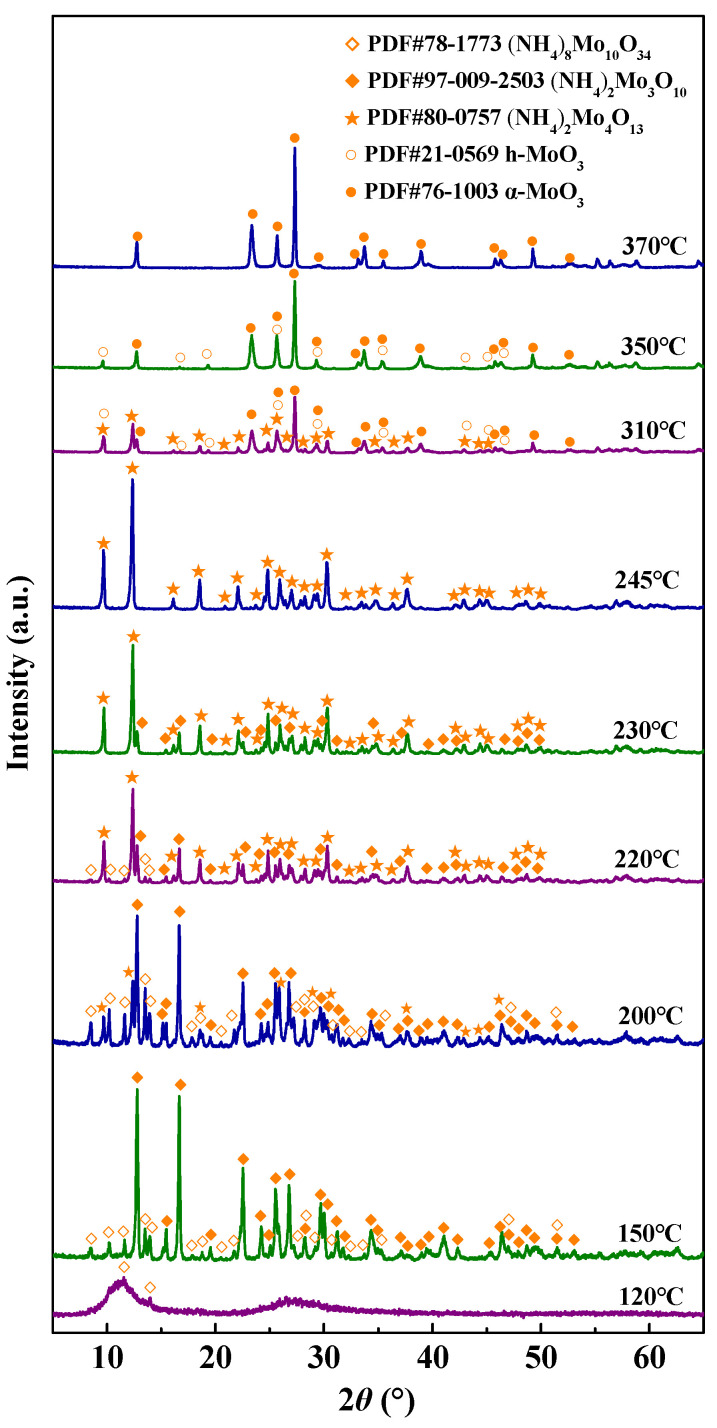
XRD patterns of the decomposition products of MoO_3_ precursor at different temperatures.

To achieve the in-depth insights of thermal decomposition behavior, the products of the MoO_3_ precursor after different decomposition temperatures for 1.5 h were determined by the X-ray diffractometer, and the XRD patterns are represented in Figure 5. Evidently, the partial crystallization peaks of (NH_4_)_8_Mo_10_O_34_ occur at 120 °C. At 150 °C, both (NH_4_)_8_Mo_10_O_34_ and (NH_4_)_2_Mo_3_O_10_ are present. However, (NH_4_)_2_Mo_4_O_13_ appears at 200 °C. At the temperature of 220 °C, the diffraction peak of (NH_4_)_8_Mo_10_O_34_ progressively weakens, while the diffraction peak of (NH_4_)_2_Mo_4_O_13_ is intensified. Subsequently, (NH_4_)_8_Mo_10_O_34_ disappears at 230 °C. When the temperature reaches 245 °C, there is only a presence of (NH_4_)_2_Mo_4_O_13_ phase. At 310 °C, there are h-MoO_3,_ α-MoO_3,_ and (NH_4_)_2_Mo_4_O_13_ phase. (NH_4_)_2_Mo_4_O_13_ phase disappears at 350 °C. At 370 °C, there is only α-MoO_3_ phase.

### 3.3. Thermal Decomposition Mechanism of MoO_3_ Precursor

Based on XRD analysis and TG-DSC/MS results, it is believed that the decomposition of MoO_3_ precursors experiences the five different stages. Firstly, in the temperature ranging from 25 °C to 182.5 °C (the endothermic peak occurred at 132.7 °C), the amorphous powder transforms into (NH_4_)_8_Mo_10_O_34_ and (NH_4_)_2_Mo_3_O_10_. The release of the water and ammonium ions results in a mass loss of 5.01% (see Figure 3). The reaction can be expressed as follows:

Amorphous (s) → x(NH_4_)_8_Mo_10_O_34_ (s) + y(NH_4_)_2_Mo_3_O_10_ (s) + mNH_3_ (g) + nH_2_O (g) (2)

Secondly, due to the small release of NH_3_ and H_2_O, there is a less mass loss (1.36%) in the range of 182.5–224.3 °C. Combined with the results reported by Kovács et al. [29], it can infer that (NH_4_)_8_Mo_10_O_34_ transforms into (NH_4_)_2_Mo_3_O_10_ during the exothermic process, which can be depicted as Equation (2).

3(NH_4_)_8_Mo_10_O_34_ (s) → 10(NH_4_)_2_Mo_3_O_10_ (s) + 4NH_3_ (g) + 2H_2_O (g) (3)

Thirdly, with the release of the ammonium ions of (NH_4_)_2_Mo_3_O_10_ at the temperature ranging from 224.3 °C to 264.5 °C, (NH_4_)_2_Mo_4_O_13_ are formed by the further loss of H_2_O and NH_3_ (Equation (4)). At the second and third decomposition stages, though (NH_4_)_8_Mo_10_O_34_ and (NH_4_)_2_Mo_3_O_10_ can decompose simultaneously in a certain temperature range, the decomposition of (NH_4_)_8_Mo_10_O_34_ finishes in advance. As a result, it is thought that the phase transformation generates from the coexistence of (NH_4_)_8_Mo_10_O_34_ and (NH_4_)_2_Mo_3_O_10_ to the disappearance of (NH_4_)_8_Mo_10_O_34_ and (NH_4_)_2_Mo_3_O_10_ in sequence, and the final formation of a single (NH_4_)_2_Mo_4_O_13_ at 245 °C.

4(NH_4_)_2_Mo_3_O_10_ (s) → 3(NH_4_)_2_Mo_4_O_13_ (s) + 2NH_3_ (g) + H_2_O (g) (4)

Fourthly, with the release of H_2_O and NH_3_ from (NH_4_)_2_Mo_4_O_13_, h-MoO_3,_ or h-MoO_3_ and α-MoO_3_ generate in the range of 264.5–341.7 °C (Equation (4)).

(NH_4_)_2_Mo_4_O_13_ (s) → 4MoO_3_ (s) + 2NH_3_ (g) + H_2_O (g) (5)

Finally, in the temperature range of 341.7–415.9 °C (the exothermic peak appeared at 391.7 °C), h-MoO_3_ is converted into α-MoO_3_, which can be expressed as follows:

h-MoO_3_ (s) → α-MoO_3_ (s) (6)

### 3.4. Thermal Decomposition Kinetics of MoO_3_ Precursor

According to Kissinger’s method, the rate of the thermal decomposition reaction can be described as follows [30,31]:

(7) $$\frac{d\alpha }{dt}=A{\left(1-\alpha \right)}^{n}\exp\left(-\frac{E}{RT}\right)$$

where *α* is the degree of transformation, *t* is time, *A* is the preexponential factor, *n* is the reaction order, *R* is the gas constant, *E* is the activation energy, and *T* is the Kelvin temperature. The peak temperature *T_m_* refers to the maximum or minimum values in DSC curves at heating rate *β* [32]. By integrating and fetching the logarithm in Equation (6), the Kissinger equation can be obtained (Equation (8)).

(8) $$\ln\left(\frac{\beta }{{T_{m}}^{2}}\right)=\ln\left(\frac{RA}{E}\right)-\frac{E}{RT_{m}}$$

**Figure 6:**
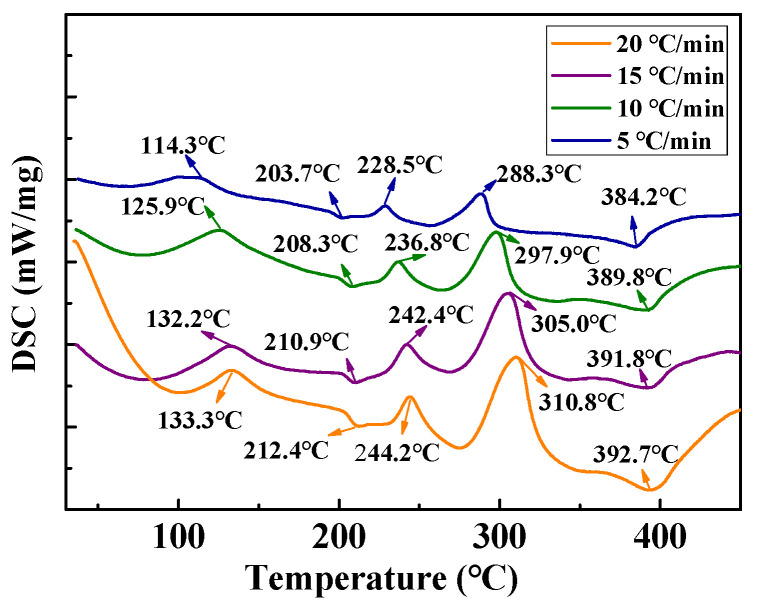
DSC curves of MoO_3_ precursor at different heating rates.

To obtain *T_m_* at different heating rates, a DSC analysis was performed on the MoO_3_ precursor at different heating rates, which are illustrated in Figure 6. Obviously, five peaks are present in all DSC curves at different heating rates, indicating reactions of (NH_4_)_8_Mo_10_O_34_, (NH_4_)_2_Mo_3_O_10_, (NH_4)2_Mo_4_O_13_, h-MoO_3,_ and α-MoO_3_. In addition, it can also be observed that the peaks shift to a higher temperature with an increase in the heating rate. This is originated from the increased thermal effect and the thermal inertia per unit time, thus causing a larger temperature difference [33,34].

**Figure 7:**
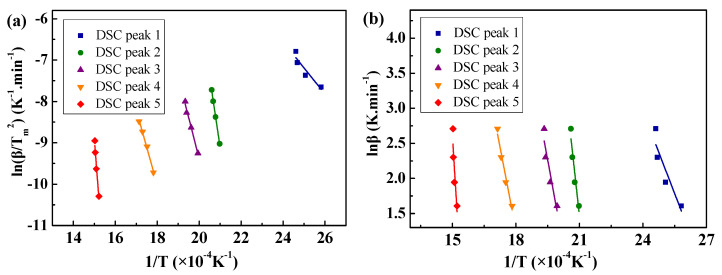
The linear fitting relation between ln(*β/T_m_*^2^) and 1/*T_m_* (**a**) and between ln*β* and 1/*T_m_* (**b**) at different heating rates.

Based on the results presented in Figure 6, the linear fitting relation between ln(*β/T_m_^2^*) and 1/*T_m_* are exhibited in Figure 7a. From Equation (8) and the linear regression equations, *E* and *A* can be, respectively, obtained by the slope −*E/R* and intercept ln(*RA/E*) (see Table 1).

Moreover, the transformation rate can also be expressed as

(9) $$\frac{E}{RT_{m}^{2}}=\frac{An}{\beta }{\left(1-\alpha \right)}^{n-1}\exp\left(-\frac{E}{RT_{m}}\right)$$

After the logarithm fetch in Equation (9), take one derivative by 1/*T_m_*, and the following equation can be obtained.

(10) $$\frac{d\left(\ln\beta \right)}{d\left(1/T_{m}\right)}=-\frac{E}{nR}$$

From the results presented in Figure 6, ln*β* − 1 displays a linear relation with *T_m_* (see Figure 7b). Based on Equation (10), the reaction orders *n* of the thermal decomposition of (NH_4_)_8_Mo_10_O_34_, (NH_4_)_2_Mo_3_O_10_, (NH_4)2_Mo_4_O_13_, h-MoO_3,_ and α-MoO_3_ can be obtained by the slope −(*E/nR*) (see Table 1). Clearly, the highest activation energy appears in the fifth peak during the thermal decomposition of the MoO_3_ precursor, corresponding to the transformation of h-MoO_3_ into α-MoO_3_. Therefore, it is thought that the dominant thermal decomposition of MoO_3_ precursor occurs during the fifth stage. The thermal decomposition rate at this stage can be represented as follows:

(11) $$\frac{d\alpha }{dt}=4.50\times 10^{43}{\left(1-\alpha \right)}^{1.33}\exp\left(-\frac{545110}{RT}\right)$$

### 3.5. Micromorphology and Properties of MoO_3_ Powder

**Figure 8:**
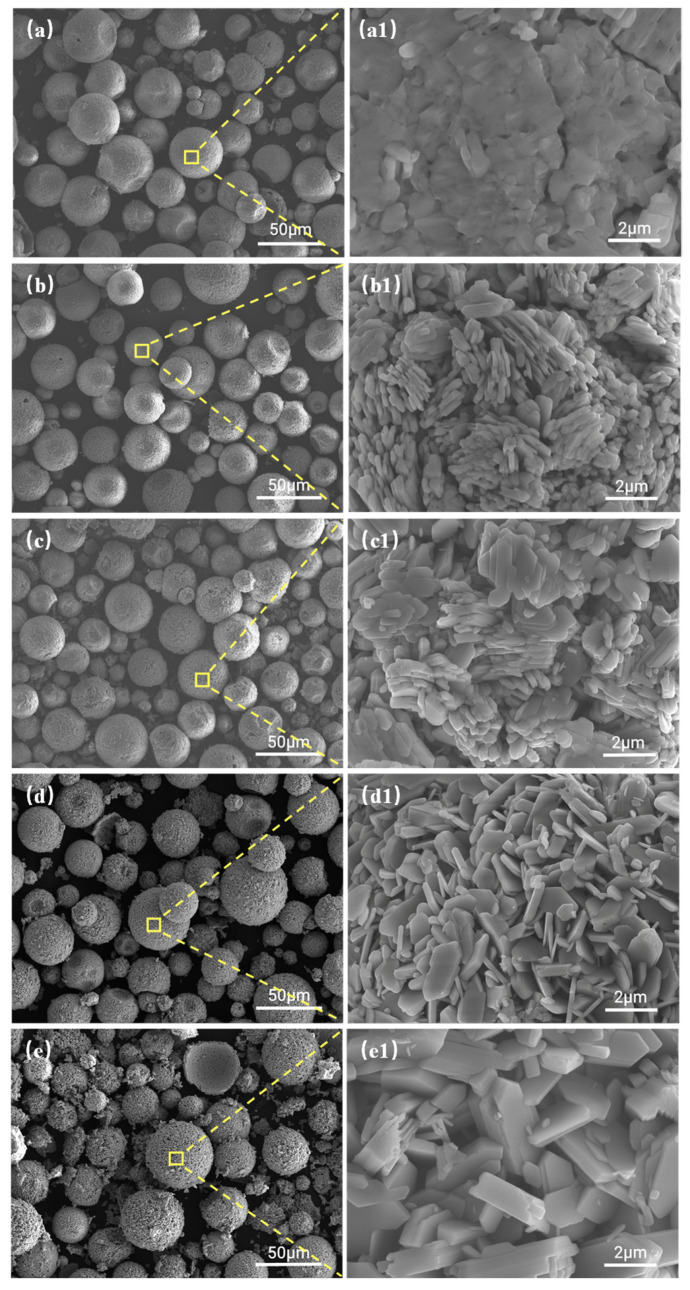
The morphologies of the MoO_3_ powders prepared at different temperatures at different magnifications: (**a**,**a1**) 370 °C, (**b**,**b1**) 400 °C, (**c**,**c1**) 450 °C, (**d**,**d1**) 500 °C, and (**e**,**e1**) 550 °C.

**Figure 9:**
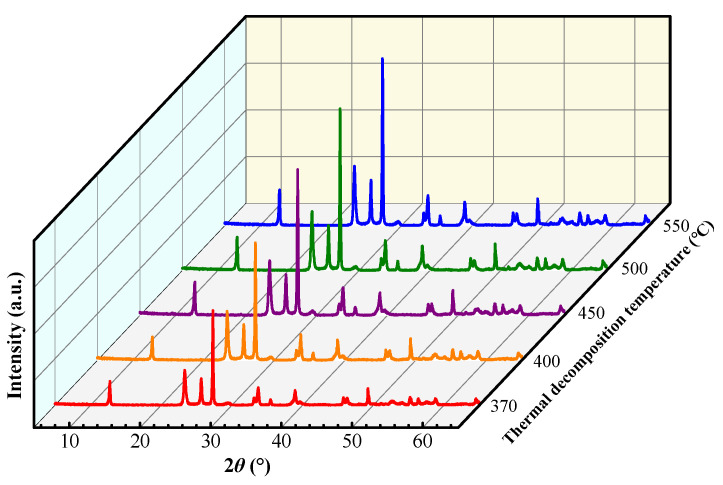
XRD patterns of MoO_3_ powders prepared at different temperatures.

Figure 8 shows the morphologies of MoO_3_ powder prepared at different thermal decomposition temperatures. Visibly, the MoO_3_ particles inherit the spherical morphology of the precursor (see Figure 2a). With an increase in temperature, the coarser surface appears together with the increased broken particles. From Figure 8(a1–e1), it can be found that a large number of fine particles aggregate into the spherical shape. At 370 °C, there are some cracks and holes on the surface of particles. Moreover, the unclear boundaries occur due to the agglomeration of fine and irregular particles (see Figure 8(a1)). At 400 °C, lots of small thin sheets overlap along with apparent boundaries (see Figure 8(b1)). From Figure 8(c1), the morphology of the reaction products at 450 °C resembles that at 400 °C (Figure 8(b1)). In Figure 8(d1), dispersed thin sheets, and thick sheets with a thickness of about 300 nm and length of about 1–3 μm at 500 °C can be observed. At 550 °C, the flake particles are piled up and grow into coarse particles with larger thickness and length (Figure 8(e1)). With an increase in decomposition temperature, the intensity of the MoO_3_ diffraction peak increases (see Figure 9), suggesting that the increased thermal decomposition temperature is beneficial for the growth of the crystal, which causes higher crystallinity and more obvious orientation, which is consistent with the change in morphologies of MoO_3_ decomposed at different temperatures.

**Figure 10:**
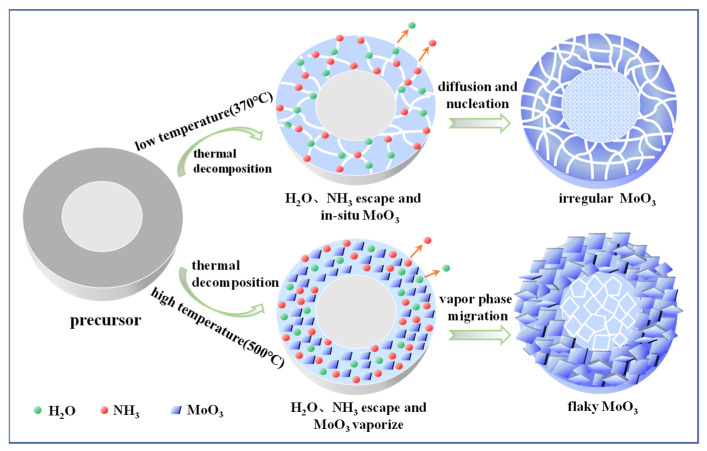
Schematic diagram illustrating the growth mechanism of MoO_3_ in the decomposition process.

As learned from the results aforementioned, prepared MoO_3_ presents different morphologies at different temperatures. To clarify the growth mechanism of MoO_3_ during the decomposition process, a schematic diagram is illustrated in Figure 10. During the thermal decomposition, the MoO_3_ precursor is decomposed into H_2_O (gas), NH_3_ (gas), and MoO_3_ (solid). With the advance of the decomposition reaction, the H_2_O and NH_3_ released from the internal precursor migrate to the outer surface, causing further decomposition until complete reaction. At a low temperature (370 °C), the stress incurred by the precursor decomposition is released by the micro-cracking, and then the in situ synthesized MoO_3_ particles nucleate and grow on the near surface of MoO_3_ reactant_._ As a result, the unclear boundary is generated by the agglomeration of fine irregular MoO_3_ particles. At 500 °C, the vapor pressure of MoO_3_ increases significantly with the temperature and hydration effect [35,36], thus causing more gasification of MoO_3_. At this time, MoO_3_ crystals form by the deposition of MoO_3_ vapor. With the further progress of the reaction, these MoO_3_ crystals progressively grow into flake morphologies. Consequently, prepared MoO_3_ displays larger sizes and high crystallinity together with well dispersion.

**Figure 11:**
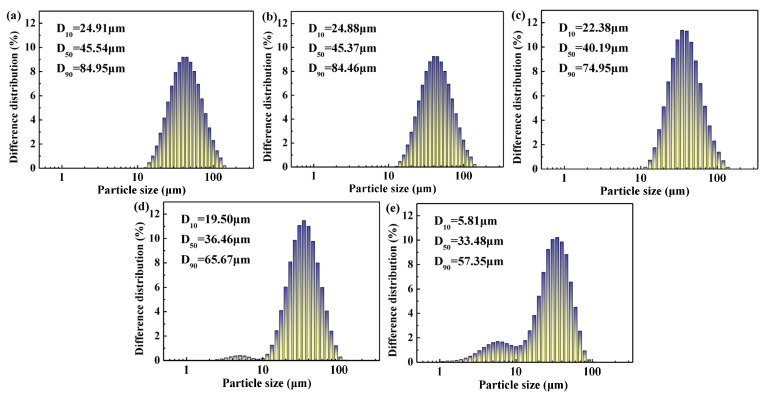
Particle size distributions of MoO_3_ prepared at different temperatures: (**a**) 370 °C, (**b**) 400 °C, (**c**) 450 °C, (**d**) 500 °C, and (**e**) 550 °C.

Figure 11 shows the particle size distributions of MoO_3_ prepared at different temperatures. It is worth noting that the D_10_, D_50,_ and D_90_ values of prepared MoO_3_ gradually reduce at different temperatures. Meanwhile, with an increase in temperature, the size distribution of the particles progressively widens and shifts toward a smaller size, implying that the particle size is reduced at a higher temperature. When the thermal decomposition temperature exceeds 500 °C, the particle size distributions present bimodal characteristics, i.e., a tiny peak appears at a small size. Moreover, partial MoO_3_ particles are broken during thermal decomposition.

**Figure 12:**
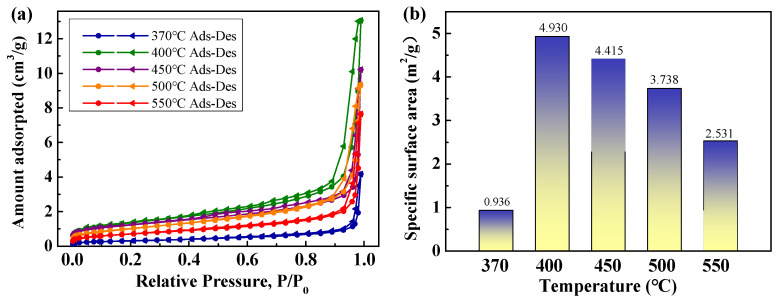
N_2_ adsorption–desorption isotherms (**a**) and specific surface area (**b**) of MoO_3_ prepared at different decomposition temperatures.

The curves of N_2_ adsorption–desorption isotherms of different MoO_3_ are presented in Figure 12a. It can be found that the N_2_ adsorption isotherms of the MoO_3_ powder are Type Ⅳ, and the hysteresis loops belong to Type H3, which indicate that the pore type is mainly slit and macroporous. Figure 12b displays the specific surface areas of MoO_3_ prepared at different temperatures. After thermal decomposition at 370 °C, it displays the less specific surface area (0.936 m^2^/g) due to the agglomeration of fine irregular particles. At 400 °C, the particles present are fine and flaky, and the specific surface area can reach up to 4.930 m^2^/g, which is larger than that of MoO_3_ prepared by spray pyrolysis (0.98m^2^/g at 400 °C, 3.09m^2^/g at 500 °C, 1.77m^2^/g at 600 °C) [19]. With a further increase in the decomposition temperature, the progressive growth of fine particles brings about a decrease in the specific surface area. Hence, it can achieve a good match of the specific surface areas and morphologies of MoO_3_ at different decomposition temperatures.

## 4. Conclusions

In this work, ultrafine flaky MoO_3_ powder is successfully synthesized using a centrifugal spray-dried precursor. It is demonstrated that there are five distinct stages during the thermal decomposition of the precursor under the environment of air. The intermediate products generate (NH_4_)_8_Mo_10_O_34_, (NH_4_)_2_Mo_3_O_10_, (NH_4_)_2_Mo_4_O_13_, h-MoO_3_, and the final product α-MoO_3_ with highly crystallinity. Moreover, the decomposition rate equation is established based on the thermal decomposition kinetic parameters of the precursor. The resulting MoO_3_ powder displays the spherical aggregates of numerous fine particles. With an increase in the decomposition temperature, the surfaces of aggregates become coarser along with an increased number of broken aggregates, and the morphology changes from unclear boundary particles to dispersed flake particles. Consequently, the fine particles exhibit larger sizes, higher crystallinity, and better dispersion, which are attributed to the mass transfer of gaseous MoO_3_ products. Additionally, the MoO_3_ particle size progressively decreases with an increase in decomposition temperature, and the specific surface area increases and then decreases. At 500 °C, it can achieve ultrafine flaky MoO_3_ powder with a thickness of about 300 nm and a length of approximate 1–3 μm, and a specific surface area of 3.738 m^2^/g.

## Figures and Tables

**Table 1:** Kinetic parameters of different reaction stages at different heating rates.

DSC Peak Sequence	The Linear Fitting Relation		*E*/(kJ/mol)	*A* (min^−1^)	*n*
	ln(*β*/*T_m_*^2^) − 1/T*_m_*	ln*β* − 1/*T_m_*			
1	y = −6324.41x + 8.62	y = −7820.61x + 21.73	52.58	3.51 × 10^7^	0.81
2	y = −34,192.96x + 62.69	y = −27,468.09x + 59.15	284.28	5.75 × 10^31^	1.24
3	y = −19,780.93x + 30.18	y = −16,457.54x + 34.35	164.46	2.53 × 10^17^	1.20
4	y = −18,061.89x + 22.49	y = −15,719.08x + 29.56	150.17	1.06 × 10^14^	1.15
5	y = −65,565.27x + 89.42	y = −49,470.59x + 76.80	545.11	4.50 × 10^43^	1.33

## Data Availability

The original contributions presented in this study are included in the article. Further inquiries can be directed to the corresponding authors.
